# Supplementary material for: Analysis of PPARγ Signaling Activity in Psoriasis
Source: Int J Mol Sci. 2021 Aug 10;22(16):8603. doi: 10.3390/ijms22168603 (PMC8395241; doi:10.3390/ijms22168603)
Supplement: Supplementary file 1 [file ijms-22-08603-s001.zip › Supplemental materials_Analysis of PPARg signaling activity in psoriasis/Pathway models/Models images and html files/Model of PPARG related pathways in psoriasis (short version)/6068.html]

NOD2


# Protein NOD2

|  |  |
| --- | --- |
| URN | urn:agi-llid:3377 |
| Total Entities | 0 |
| Connectivity | 2171 |
| Name | NOD2 |
| Description | nucleotide binding oligomerization domain containing 2 |
| Notes | This gene is a member of the Nod1/Apaf-1 family and encodes a protein with two caspase recruitment (CARD) domains and six leucine-rich repeats (LRRs). The protein is primarily expressed in the peripheral blood leukocytes. It plays a role in the immune response to intracellular bacterial lipopolysaccharides (LPS) by recognizing the muramyl dipeptide (MDP) derived from them and activating the NFKB protein. Mutations in this gene have been associated with Crohn disease and Blau syndrome. Alternatively spliced transcript variants encoding distinct isoforms have been found for this gene. [provided by RefSeq, Jun 2014] |
| ObjectType | Protein |

---

|  |  |
| --- | --- |
| Pathway | NOD-like Receptors |
|  | Crohn's Disease |
|  | Paneth-Cell Function in Crohn's Disease |
|  | Macrophage Dysfunction |
|  | Proteins Involved in Inflammatory Bowel Diseases |
|  | Genes with Susceptibility for Crohn's Disease and Ulcerative Colitis |
|  | Dendritic Cell Dysfunction in Crohn's disease |
|  | Dendritic Cell Function in Ulcerative Colitis |
|  | Proteins Involved in Ulcerative Colitis |
|  | Proteins Involved in Psoriatic Arthritis |
|  | Proteins Involved in Periodontitis |
|  | Blau Syndrome |
|  | Proteins Involved in Chronic Obstructive Pulmonary Disease |
|  | TLR1 -> 2/6 Expression Targets |
|  | TLR4 -> NF-kB/IRF Expression Targets |
|  | Model of PPARG signaling in psoriasis |
|  | Model of PPARG related pathways in psoriasis (short version) |
|  | Overview. Diseases of the digestive system (chapter 10) |
|  | 1\_Insufficient activation of immune response in the middle ear epithelium cells in otitis media |
|  | 3\_Role of alveolar macrophages in pulmonary tissue destruction in COPD |
|  | 1\_Th2 cell response in asthma |
|  | 2\_Paneth cell dysfunction in Crohn's disease |
|  | 1\_Defects in response to pathogens in the gut promotes inflammation in Crohn's disease |
|  | 3\_Autophagy dysfunction in Crohn's disease |
|  | 2\_Intestine epithelial cells drive immunological responses in ulcerative colitis |
|  | 4\_1\_Polymorphisms associated with inflammatory bowel diseases |
|  | 1\_Antigen-presenting cells promote islet dysfunction and immune system activation in T1D |

---

|  |  |
| --- | --- |
| Group | Genes Associated with Systemic Lupus Erythematosus |
|  | Genes with Mutations Associated with Crohn's Disease |
|  | Genes with Mutations Associated with Psoriatic Arthritis |

---

|  |  |
| --- | --- |
| MedScan ID | 3377 |

---

|  |  |
| --- | --- |
| LocusLink ID | 3377 |
|  | 64127 |
|  | 257632 |
|  | 291912 |

---

|  |  |
| --- | --- |
| Alias | NOD2 signaling adaptor protein |
|  | D230007K08 |
|  | inflammatory bowel disease protein I |
|  | caspase recruitment domain family, member 15 |
|  | PSORAS1 |
|  | inflammatory bowel disease 1 locus |
|  | nucleotide-binding oligomerization domain 2 |
|  | caspase recruitment domain-containing protein 15 |
|  | F830032C23Rik |
|  | caspase recruitment domains 15 |
|  | NOD2 |
|  | nucleotide-binding oligomerization domain, leucine rich repeat and CARD domain containing 2 |
|  | CLR16.3 |
|  | YAOS |
|  | NLR family, CARD domain containing 2 |
|  | nucleotide-binding oligomerization domain II |
|  | inflammatory bowel disease 1 |
|  | RIKEN cDNA F830032C23 gene |
|  | OTTHUMP00000164224 |
|  | Caspase-Activation Recruitment Domain 15 |
|  | nucleotide-binding oligomerization domain containing 2 |
|  | inflammatory bowel disease locus 1 |
|  | PSORAS I |
|  | inflammatory bowel disease protein 1 |
|  | OTTMUSP00000038424 |
|  | Nlrc2 |
|  | IBD I |
|  | inflammatory bowel disease protein-1 |
|  | F830032C23 |
|  | IBD1 |
|  | IBD1 locus |
|  | NOD-like receptor C2 |
|  | Psoriatic arthritis, susceptibility to, 1 |
|  | CARD15 |
|  | Psoriatic arthritis, susceptibility to, I |
|  | BLAU |
|  | Caspase-Activation Recruitment Domain containing protein 15 |
|  | Arthrocutaneouveal granulomatosis (Blau syndrome) gene |
|  | Inflammatory bowel disease-1 (Crohn disease) locus |
|  | nucleotide oligomerization domain protein 2 |
|  | nod2b |
|  | CD |
|  | ACUG |
|  | BLAUS |
|  | caspase recruitment domain protein 15 |
|  | nucleotide-binding oligomerization domain-containing protein 2 |
|  | D230007K08Rik |
|  | Card15\_predicted |

---

|  |  |
| --- | --- |
| GO ID | 0005524 |
|  | 0050700 |
|  | 0030544 |
|  | 0051879 |
|  | 0003779 |
|  | 0019899 |
|  | 0032500 |
|  | 0042834 |
|  | 0019901 |
|  | 0044877 |
|  | 0007254 |
|  | 0000187 |
|  | 0071222 |
|  | 0071225 |
|  | 0071407 |
|  | 0071224 |
|  | 0002367 |
|  | 0002374 |
|  | 0006952 |
|  | 0042742 |
|  | 0016045 |
|  | 0009595 |
|  | 0032498 |
|  | 0045087 |
|  | 0070498 |
|  | 0035556 |
|  | 0030277 |
|  | 2000110 |
|  | 0070431 |
|  | 0070423 |
|  | 0050871 |
|  | 0070374 |
|  | 0043123 |
|  | 0046330 |
|  | 0043406 |
|  | 0051092 |
|  | 1901224 |
|  | 0045747 |
|  | 1900017 |
|  | 0002606 |
|  | 0002732 |
|  | 0050679 |
|  | 0046645 |
|  | 0032731 |
|  | 0050718 |
|  | 0032733 |
|  | 0032740 |
|  | 0032755 |
|  | 0032757 |
|  | 0051770 |
|  | 0051353 |
|  | 0043552 |
|  | 2000363 |
|  | 0060585 |
|  | 1902523 |
|  | 0032874 |
|  | 0045944 |
|  | 0032760 |
|  | 0002830 |
|  | 0051259 |
|  | 0050727 |
|  | 0032495 |
|  | 0007584 |
|  | 0005794 |
|  | 0016323 |
|  | 0009986 |
|  | 0005737 |
|  | 0005856 |
|  | 0005829 |
|  | 0005739 |
|  | 0005886 |
|  | 0032991 |
|  | 0031982 |
|  | 0016888 |
|  | 0000166 |
|  | 0002253 |
|  | 0050830 |
|  | 0002376 |
|  | 0002381 |
|  | 0006954 |
|  | 0002227 |
|  | 0071608 |
|  | 0032088 |
|  | 0002710 |
|  | 0044130 |
|  | 0002862 |
|  | 0032689 |
|  | 0032695 |
|  | 0032701 |
|  | 0032703 |
|  | 0034136 |
|  | 0032720 |
|  | 0043410 |
|  | 0006963 |
|  | 0006965 |
|  | 0008284 |
|  | 0002925 |
|  | 0045089 |
|  | 0032735 |
|  | 0071639 |
|  | 0050731 |
|  | 0050766 |
|  | 0031398 |
|  | 1904417 |
|  | 0042981 |
|  | 0090022 |
|  | 0043330 |
|  | 0032496 |
|  | 0032494 |
|  | 0007165 |
|  | 0016020 |
|  | 0035419 |
|  | 0008180 |
|  | 0043234 |
|  | 0002755 |
|  | 0002756 |
|  | 0035666 |
|  | 0035872 |
|  | 0051403 |
|  | 0034166 |
|  | 0034134 |
|  | 0034138 |
|  | 0034142 |
|  | 0034146 |
|  | 0034162 |
|  | 0038123 |
|  | 0038124 |
|  | 0002224 |
|  | 0005622 |
|  | 0008063 |
|  | 0034130 |
|  | 0002282 |
|  | 0052033 |
|  | 0008152 |
|  | 0042995 |

---

|  |  |
| --- | --- |
| KEGG ID | hsa:64127 |
|  | mmu:257632 |
|  | rno:291912 |

---

|  |  |
| --- | --- |
| Organism | Homo sapiens {Organism urn:agi-taxid:9606} |
|  | Mus musculus {Organism urn:agi-taxid:10090} |
|  | Rattus norvegicus {Organism urn:agi-taxid:10116} |
|  | Homo sapiens |
|  | Mus musculus |
|  | Rattus norvegicus |

---

|  |  |
| --- | --- |
| Mouse chromosome position | 8 |

---

|  |  |
| --- | --- |
| OMIM ID | 605956 |
|  | 186580 |
|  | 266600 |
|  | 607507 |
|  | 617321 |
|  | 609464 |

---

|  |  |
| --- | --- |
| Rat chromosome position | 19p11 |

---

|  |  |
| --- | --- |
| Hugo ID | 5331 |
|  | HGNC:5331 |

---

|  |  |
| --- | --- |
| Human chromosome position | 16q12.1 |
|  | 16q21 |

---

|  |  |
| --- | --- |
| Swiss-Prot Accession | Q9HC29 |
|  | A0A286YF65 |
|  | Q9HC29.1 |
|  | Q80SY9 |
|  | Q8K3Z0.1 |
|  | D4A5W3 |
|  | E2JEQ6 |
|  | Q96RH5 |
|  | Q96RH6 |
|  | Q96RH8 |
|  | Q8K3Z0 |
|  | Q6TDC3 |
|  | Q7Z595 |
|  | Q7Z596 |
|  | Q7Z597 |
|  | Q2PZA2 |
|  | Q2PZA3 |
|  | Q3V2V4 |
|  | Q3V2V8 |
|  | Q80SS7 |
|  | Q80SV8 |
|  | Q80SY7 |
|  | Q80SY8 |
|  | Q80SZ8 |
|  | Q80UH1 |
|  | Q80UH3 |
|  | Q80UH4 |
|  | Q80UH5 |
|  | Q80UH6 |
|  | Q80UH7 |

---

|  |  |
| --- | --- |
| GenBank ID | NC\_000016 |
|  | NR\_163434 |
|  | XR\_933387 |
|  | XR\_429725 |
|  | XR\_429726 |
|  | XM\_017023536 |
|  | XP\_016879025 |
|  | XM\_017023537 |
|  | XP\_016879026 |
|  | NM\_001370466 |
|  | NP\_001357395 |
|  | XM\_006721242 |
|  | XP\_006721305 |
|  | XM\_011523260 |
|  | XP\_011521562 |
|  | XM\_006721243 |
|  | XP\_006721306 |
|  | XM\_011523261 |
|  | XP\_011521563 |
|  | NM\_022162 |
|  | NP\_071445 |
|  | NM\_001293557 |
|  | NP\_001280486 |
|  | XM\_011523259 |
|  | XP\_011521561 |
|  | XM\_017023538 |
|  | XP\_016879027 |
|  | XM\_017023535 |
|  | XP\_016879024 |
|  | NG\_007508 |
|  | AB542700 |
|  | BAJ13469 |
|  | AB542701 |
|  | BAJ13470 |
|  | AC007608 |
|  | AC007728 |
|  | AF385085 |
|  | AAK70863 |
|  | AF385086 |
|  | AAK70864 |
|  | AF385087 |
|  | AAK70865 |
|  | AF385088 |
|  | AAK70866 |
|  | AF385089 |
|  | AAK70867 |
|  | AF385090 |
|  | AAK70868 |
|  | AF538972 |
|  | AJ303140 |
|  | CAC42117 |
|  | AX259774 |
|  | CAD10212 |
|  | AX259776 |
|  | CAD10248 |
|  | CH471092 |
|  | EAW82767 |
|  | DQ869189 |
|  | ABI96990 |
|  | EF364441 |
|  | ABN42698 |
|  | EF624392 |
|  | ABS17610 |
|  | EF624393 |
|  | ABS17611 |
|  | EF624394 |
|  | ABS17612 |
|  | EU817180 |
|  | ACF33179 |
|  | EU888272 |
|  | ACF95817 |
|  | EU888273 |
|  | ACF95818 |
|  | EU888274 |
|  | ACF95819 |
|  | EU888275 |
|  | ACF95820 |
|  | FJ755457 |
|  | ACX94233 |
|  | FN651786 |
|  | CBJ18719 |
|  | FN651787 |
|  | FN651788 |
|  | MG581415 |
|  | AYE92798 |
|  | AF178930 |
|  | AAG33677 |
|  | AK298099 |
|  | BAG60384 |
|  | AY187233 |
|  | AY187234 |
|  | AY187235 |
|  | AY187236 |
|  | AY187237 |
|  | AY187238 |
|  | AY187239 |
|  | AY187240 |
|  | AY187241 |
|  | AY187242 |
|  | AY187243 |
|  | AY187244 |
|  | AY187245 |
|  | AY187246 |
|  | AY423901 |
|  | AAQ93806 |
|  | DQ868973 |
|  | ABJ16061 |
|  | HQ204571 |
|  | ADN95581 |
|  | HY272594 |
|  | Q9HC29 |
|  | NC\_000074 |
|  | XM\_011248390 |
|  | XP\_011246692 |
|  | XM\_006531029 |
|  | XP\_006531092 |
|  | NM\_145857 |
|  | NP\_665856 |
|  | XM\_006531028 |
|  | XP\_006531091 |
|  | AC156937 |
|  | AH012056 |
|  | AAN52480 |
|  | AH012057 |
|  | AAN52481 |
|  | AH012058 |
|  | AAN52482 |
|  | AH012059 |
|  | AAN52483 |
|  | AH012060 |
|  | AAN52484 |
|  | AH012062 |
|  | AAN52934 |
|  | AH012063 |
|  | AAN52935 |
|  | AH012064 |
|  | AAN52936 |
|  | AH012065 |
|  | AAN52937 |
|  | AH012067 |
|  | AAN52939 |
|  | AH012069 |
|  | AAN57787 |
|  | AH012070 |
|  | AAN57788 |
|  | AH012071 |
|  | AAN57789 |
|  | AH012072 |
|  | AAN57790 |
|  | AH012073 |
|  | AAN57791 |
|  | AH012074 |
|  | AAN57792 |
|  | AH012075 |
|  | AAN57793 |
|  | AH012076 |
|  | AAN59991 |
|  | AH012077 |
|  | AAN59992 |
|  | AH012078 |
|  | AAN59993 |
|  | AH012079 |
|  | AAN59994 |
|  | AH012080 |
|  | AAN59995 |
|  | AH012104 |
|  | AAN60733 |
|  | AH012105 |
|  | AAN60734 |
|  | AH012106 |
|  | AAN60735 |
|  | AH012107 |
|  | AAN60736 |
|  | AH012108 |
|  | AAN60737 |
|  | AH012111 |
|  | AAN62467 |
|  | AH012112 |
|  | AAN62468 |
|  | AH012113 |
|  | AAN62469 |
|  | AH012114 |
|  | AAN62470 |
|  | AH012115 |
|  | AAN62471 |
|  | AH012116 |
|  | AAN62472 |
|  | AH012151 |
|  | AAN63034 |
|  | AH012152 |
|  | AAN63035 |
|  | AH012153 |
|  | AAN63036 |
|  | AH012154 |
|  | AAN63037 |
|  | AH012155 |
|  | AAN63038 |
|  | AH012156 |
|  | AAN63039 |
|  | AH012157 |
|  | AAN63040 |
|  | AH012158 |
|  | AAN63041 |
|  | AH012198 |
|  | AAN78329 |
|  | AH012199 |
|  | AAN78330 |
|  | AH012203 |
|  | AAN84594 |
|  | CAAA01040580 |
|  | CH466525 |
|  | EDL11059 |
|  | AF520774 |
|  | AAM76073 |
|  | AK089390 |
|  | BAE43397 |
|  | AK089843 |
|  | BAE43401 |
|  | AY160220 |
|  | AAN52477 |
|  | AY160221 |
|  | AAN52478 |
|  | BC044774 |
|  | AAH44774 |
|  | DQ289012 |
|  | ABC25455 |
|  | DQ289013 |
|  | ABC25456 |
|  | Q8K3Z0 |
|  | NC\_005118 |
|  | NM\_001106172 |
|  | NP\_001099642 |
|  | XM\_017601207 |
|  | XP\_017456696 |
|  | XM\_008772372 |
|  | XP\_008770594 |
|  | XM\_008772371 |
|  | XP\_008770593 |
|  | XM\_008772373 |
|  | XP\_008770595 |
|  | AC\_000087 |
|  | AABR07043076 |
|  | AAHX01097535 |
|  | AAHX01097536 |
|  | AAHX01097537 |
|  | AAHX01097538 |
|  | AAHX01097539 |
|  | AAHX01097540 |
|  | CH474037 |
|  | EDL87529 |
|  | XM\_005256084 |
|  | XP\_005256141 |
|  | XM\_011523258 |
|  | XP\_011521560 |
|  | XM\_011523257 |
|  | XP\_011521559 |
|  | NC\_018927 |
|  | AMYH02032016 |
|  | HG508052 |
|  | BC152737 |
|  | AAI52738 |
|  | BC156571 |
|  | AAI56572 |
|  | AC\_000030 |
|  | AAHY01072987 |
|  | AAHY01072988 |
|  | AAHY01072989 |
|  | AAHY01072990 |
|  | AY160229 |
|  | AY160230 |
|  | AY160231 |
|  | AY160232 |
|  | AY160233 |
|  | AY160234 |
|  | AY160235 |
|  | AY160236 |
|  | AY160237 |
|  | AY160238 |
|  | AY160239 |
|  | AY160240 |
|  | AY160241 |
|  | AY160242 |
|  | AY160243 |
|  | AY160244 |
|  | AY160245 |
|  | AY160246 |
|  | AY160247 |
|  | AY160248 |
|  | AY160249 |
|  | AY160250 |
|  | AY160251 |
|  | AY160252 |
|  | AY160253 |
|  | AY160254 |
|  | AY160255 |
|  | AY160256 |
|  | AY160257 |
|  | AY160258 |
|  | AY160259 |
|  | AY160260 |
|  | AY160261 |
|  | AY160262 |
|  | AY160263 |
|  | AY160264 |
|  | AY160265 |
|  | AY160266 |
|  | AY160267 |
|  | AY160268 |
|  | AY160269 |
|  | AY160270 |
|  | AY160271 |
|  | AY160272 |
|  | AY160273 |
|  | AY160274 |
|  | AY160275 |
|  | AY160276 |
|  | AY160277 |
|  | AY160278 |
|  | AY160279 |
|  | AY160280 |
|  | AY160281 |
|  | AY160282 |
|  | AY160283 |
|  | AY160284 |
|  | AY160285 |
|  | AY160286 |
|  | AY160287 |
|  | AY160288 |
|  | AY160289 |
|  | AY160290 |
|  | AY160291 |
|  | AY160292 |
|  | AY160293 |
|  | AY160294 |
|  | AY160295 |
|  | AY160296 |
|  | AY160297 |
|  | AY160298 |
|  | AY160299 |
|  | AY160300 |
|  | AY160301 |
|  | AY160302 |
|  | AY160303 |
|  | AY160304 |
|  | AY160305 |
|  | AY160306 |
|  | AY160307 |
|  | AY160308 |
|  | AY160309 |
|  | AY160310 |
|  | AY160311 |
|  | AY160312 |
|  | AY160313 |
|  | AY160314 |
|  | AY160315 |
|  | AY160316 |
|  | AY160317 |
|  | AY160318 |
|  | AY160329 |
|  | AY160330 |
|  | AY160331 |
|  | AY160332 |
|  | AY160333 |
|  | AY160334 |
|  | AY160335 |
|  | AY160336 |
|  | AY160337 |
|  | AY160338 |
|  | AY160339 |
|  | AY160340 |
|  | AY160341 |
|  | AY160342 |
|  | AY160343 |
|  | AY160344 |
|  | AY160345 |
|  | AY160346 |
|  | AY160347 |
|  | AY160348 |
|  | AY160349 |
|  | AY160350 |
|  | AY160351 |
|  | AY160352 |
|  | AY160353 |
|  | AY160354 |
|  | AY160355 |
|  | AY160356 |
|  | AY160357 |
|  | AY160358 |
|  | AY160359 |
|  | AY160360 |
|  | AY160361 |
|  | AY160362 |
|  | AY160363 |
|  | AY160364 |
|  | AY160365 |
|  | AY160366 |
|  | AY160367 |
|  | AY160368 |
|  | AY160369 |
|  | AY160370 |
|  | AY160371 |
|  | AY160372 |
|  | AY160373 |
|  | AY160374 |
|  | AY160375 |
|  | AY160376 |
|  | AY160377 |
|  | AY160378 |
|  | AY160379 |
|  | AY160380 |
|  | AY160381 |
|  | AY160382 |
|  | AY160383 |
|  | AY160384 |
|  | AY160385 |
|  | AY160386 |
|  | AY160387 |
|  | AY160388 |
|  | AY160389 |
|  | AY160390 |
|  | AY160391 |
|  | AY160392 |
|  | AY160393 |
|  | AY160394 |
|  | AY160395 |
|  | AY160396 |
|  | AY160397 |
|  | AY160398 |
|  | AY160399 |
|  | AY160400 |
|  | AY160401 |
|  | AY160402 |
|  | AY160403 |
|  | AY160404 |
|  | AY160405 |
|  | AY160406 |
|  | AY160407 |
|  | AY160408 |
|  | AY160409 |
|  | AY160410 |
|  | AY160411 |
|  | AY160412 |
|  | AY160413 |
|  | AY160414 |
|  | AY160415 |
|  | AY160416 |
|  | AY160417 |
|  | AY160418 |
|  | AY160419 |
|  | AY160420 |
|  | AY160421 |
|  | AY160422 |
|  | AY160423 |
|  | AY160424 |
|  | AY160425 |
|  | AY160426 |
|  | AY160427 |
|  | AY160428 |
|  | AY160429 |
|  | AY160430 |
|  | AY160431 |
|  | AY160432 |
|  | AY160433 |
|  | AY160434 |
|  | AY160435 |
|  | AY160436 |
|  | AY160437 |
|  | AY160438 |
|  | AY160439 |
|  | AY160440 |
|  | AY160441 |
|  | AY160442 |
|  | AY160443 |
|  | AY160444 |
|  | AY160445 |
|  | AY160446 |
|  | AY160447 |
|  | AY160448 |
|  | AY160449 |
|  | AY160450 |
|  | AY160451 |
|  | AY160452 |
|  | AY160453 |
|  | AY160454 |
|  | AY160455 |
|  | AY160456 |
|  | AY160457 |
|  | AY160458 |
|  | AY160459 |
|  | AY160460 |
|  | AY160461 |
|  | AY160462 |
|  | AY160463 |
|  | AY160464 |
|  | AY160465 |
|  | AY160466 |
|  | AY160467 |
|  | AY160468 |
|  | AY160469 |
|  | AY160470 |
|  | AY160471 |
|  | AY160472 |
|  | AY160473 |
|  | AY160474 |
|  | AY160475 |
|  | AY160476 |
|  | AY160477 |
|  | AY160478 |
|  | AY160479 |
|  | AY160480 |
|  | AY160481 |
|  | AY160482 |
|  | AY160483 |
|  | AY160484 |
|  | AY160485 |
|  | AY160486 |
|  | AY160487 |
|  | AY160488 |
|  | AY160489 |
|  | AY160490 |
|  | AY160491 |
|  | AY160492 |
|  | AY160493 |
|  | AY160494 |
|  | AY160495 |
|  | AY160496 |
|  | AY160497 |
|  | AY160498 |
|  | AY160499 |
|  | AY160500 |
|  | AY160501 |
|  | AY160502 |
|  | AY160503 |
|  | AY160504 |
|  | AY160505 |
|  | AY160506 |
|  | AY160507 |
|  | AY160508 |
|  | AY160509 |
|  | AY160510 |
|  | AY160511 |
|  | AY160512 |
|  | AY160513 |
|  | AY160514 |
|  | AY160515 |
|  | AY160516 |
|  | AY160517 |
|  | AY160518 |
|  | AY160519 |
|  | AY160520 |
|  | AY160521 |
|  | AY160522 |
|  | AY160523 |
|  | AY160524 |
|  | AY160525 |
|  | AY160526 |
|  | AY160527 |
|  | AY160528 |
|  | AY160529 |
|  | AY160530 |
|  | AY160531 |
|  | AY160532 |
|  | AY160533 |
|  | AY160534 |
|  | AY160535 |
|  | AY160536 |
|  | AY160537 |
|  | AY160538 |
|  | AY160539 |
|  | AY160540 |
|  | AY160541 |
|  | AY160542 |
|  | AY160543 |
|  | AY160544 |
|  | AY160545 |
|  | AY160546 |
|  | AY160547 |
|  | AY160548 |
|  | AY160549 |
|  | AY160550 |
|  | AY160551 |
|  | AY160552 |
|  | AY160553 |
|  | AY160554 |
|  | AY160555 |
|  | AY160556 |
|  | AY160557 |
|  | AY160558 |
|  | AY160559 |
|  | AY160560 |
|  | AY160561 |
|  | AY160562 |
|  | AY160563 |
|  | AY160564 |
|  | AY160565 |
|  | AY160566 |
|  | AY160567 |
|  | AY160568 |
|  | AY160569 |
|  | AY160570 |
|  | AY160571 |
|  | AY160572 |
|  | AY160573 |
|  | AY160574 |
|  | AY160575 |
|  | AY160576 |
|  | AY160577 |
|  | AY160578 |
|  | AY160579 |
|  | AY160580 |
|  | AY160581 |
|  | AY160582 |
|  | AY160583 |
|  | AY160584 |
|  | AY160585 |
|  | AY160586 |
|  | AY160587 |
|  | AY160588 |
|  | AY160589 |
|  | AY160590 |
|  | AY160591 |
|  | AY160592 |
|  | AY160593 |
|  | AY160594 |
|  | AY160595 |
|  | AY160596 |
|  | AY160597 |
|  | AY160598 |
|  | AY160599 |
|  | AY160600 |
|  | AY160601 |
|  | AY160602 |
|  | AY160603 |
|  | AY160604 |
|  | AY160605 |
|  | AY160606 |
|  | AY160607 |
|  | AY160608 |
|  | AY160609 |
|  | AY160610 |
|  | AY160611 |
|  | AY160612 |
|  | AY160613 |
|  | AY160614 |
|  | AY160615 |
|  | AY160616 |
|  | AY160617 |
|  | AY160618 |
|  | AY160619 |
|  | AY160620 |
|  | AY160621 |
|  | AY160622 |
|  | AY160623 |
|  | AY160624 |
|  | AY160625 |
|  | AY160626 |
|  | AY160627 |
|  | AY160628 |
|  | AY160629 |
|  | AY160630 |
|  | AY160631 |
|  | AY160632 |
|  | AY160633 |
|  | AY160634 |
|  | AY160635 |
|  | AY160636 |
|  | AY160637 |
|  | AY160638 |
|  | AY160639 |
|  | AY160640 |
|  | AY160641 |
|  | AY160642 |
|  | AY160643 |
|  | AY160644 |
|  | AY160645 |
|  | AY160646 |
|  | AY160647 |
|  | AY160648 |
|  | AY160649 |
|  | AY160650 |
|  | AY160651 |
|  | AY160652 |
|  | AY160653 |
|  | AY160654 |
|  | AY160655 |
|  | AY160656 |
|  | AY160657 |
|  | AY160658 |
|  | AY160659 |
|  | AY160660 |
|  | AY160661 |
|  | AY160662 |
|  | AY160663 |
|  | AY160664 |
|  | AY160665 |
|  | AY160666 |
|  | AY160667 |
|  | AY160668 |
|  | AY160669 |
|  | AY160670 |
|  | AY160671 |
|  | AY160672 |
|  | AY160673 |
|  | AY160674 |
|  | AY160675 |
|  | AY160676 |
|  | AY160677 |
|  | AY160678 |
|  | BC168407 |
|  | AAI68407 |
|  | XM\_008772370 |
|  | XP\_008770592 |
|  | AC\_000148 |
|  | ABBA01000867 |
|  | XM\_006531027 |
|  | XP\_006531090 |
|  | NT\_010498 |
|  | NW\_926462 |
|  | NW\_001838288 |
|  | AC\_000059 |
|  | Q6TDC3 |
|  | Q7Z595 |
|  | Q7Z596 |
|  | Q7Z597 |
|  | NT\_078575 |
|  | NW\_001030904 |
|  | Q2PZA2 |
|  | Q2PZA3 |
|  | Q3V2V4 |
|  | Q3V2V8 |
|  | Q80SS7 |
|  | Q80SV8 |
|  | Q80SY7 |
|  | Q80SY8 |
|  | Q80SY9 |
|  | Q80SZ8 |
|  | Q80UH1 |
|  | Q80UH3 |
|  | Q80UH4 |
|  | Q80UH5 |
|  | Q80UH6 |
|  | Q80UH7 |
|  | NW\_047534 |
|  | NW\_001084743 |

---

|  |  |
| --- | --- |
| Swiss-Prot ID | NOD2\_HUMAN |
|  | Q80SY9\_MOUSE |
|  | D4A5W3\_RAT |

---

|  |  |
| --- | --- |
| Cell Localization | Cytoplasm |
|  | Membrane |
|  | Mitochondrion |
|  | Basolateral cell membrane |

---

|  |  |
| --- | --- |
| Ensembl ID | ENSG00000167207 |
|  | ENSP00000300589.2 |
|  | ENST00000300589.6 |
|  | ENSP00000496533.1 |
|  | ENST00000646677.1 |
|  | ENSP00000493088.1 |
|  | ENST00000641284.1 |
|  | ENSMUSG00000055994 |
|  | ENSMUSP00000113773.1 |
|  | ENSMUST00000118370.7 |
|  | ENSRNOG00000014124 |
|  | ENSRNOP00000061463.1 |
|  | ENSRNOT00000065103.2 |
|  | ENSP00000300589 |
|  | ENST00000300589 |
|  | ENSMUSP00000113773 |
|  | ENSMUST00000118370 |
|  | ENSRNOP00000061463 |
|  | ENSRNOT00000065103 |

---

|  |  |
| --- | --- |
| MGI ID | MGI:2429397 |
|  | 2429397 |

---

|  |  |
| --- | --- |
| RGD ID | 1306368 |

---

|  |  |
| --- | --- |
| Unigene ID | Hs.592072 |
|  | Mm.222633 |
|  | Rn.218600 |

---

|  |  |
| --- | --- |
| Homologene ID | 11156 |

---

|  |  |
| --- | --- |
| IPI ID | IPI00005559 |
|  | IPI00759531 |
|  | IPI00886252 |
|  | IPI00948526 |
|  | IPI00230403 |
|  | IPI00223373 |
|  | IPI00720136 |
|  | IPI00359351 |

---
